# Risk of newly diagnosed interstitial lung disease after COVID-19 and impact of vaccination: a nationwide population-based cohort study

**DOI:** 10.3389/fpubh.2023.1295457

**Published:** 2024-01-08

**Authors:** Bo-Guen Kim, Hyun Lee, Cho Yun Jeong, Sang Woo Yeom, Dong Won Park, Tai Sun Park, Ji-Yong Moon, Tae-Hyung Kim, Jang Won Sohn, Ho Joo Yoon, Jong Seung Kim, Sang-Heon Kim

**Affiliations:** ^1^Department of Internal Medicine, Hanyang University College of Medicine, Seoul, Republic of Korea; ^2^Department of Medical Informatics, Jeonbuk National University Medical School, Jeonju, Republic of Korea; ^3^Research Institute of Clinical Medicine of Jeonbuk National University—Biomedical Research Institute of Jeonbuk National University Hospital, Jeonju, Republic of Korea; ^4^Department of Otorhinolaryngology-Head and Neck Surgery, Jeonbuk National University Medical School, Jeonju, Republic of Korea

**Keywords:** COVID-19, COVID-19 vaccination, interstitial lung disease, risk, epidemiology

## Abstract

**Objectives:**

Previous studies suggested that coronavirus disease 2019 (COVID-19) could lead to pulmonary fibrosis, but the incidence of newly diagnosed interstitial lung disease (ILD) after COVID-19 is unclear. We aimed to determine whether COVID-19 increases the risk of newly diagnosed ILD and whether vaccination against COVID-19 can reduce this risk.

**Methods:**

This retrospective cohort study used data from the Korean National Health Insurance claim-based database. Two study groups and propensity score (PS)-matched control groups were constructed: Study 1: participants diagnosed with COVID-19 (COVID-19 cohort) and their PS-matched controls; Study 2: COVID-19 vaccinated participants (vaccination cohort) and their PS-matched controls.

**Results:**

In Study 1, during a median 6 months of follow-up, 0.50% of the COVID-19 cohort (300/60,518) and 0.04% of controls (27/60,518) developed newly diagnosed ILD, with an incidence of 9.76 and 0.88 per 1,000 person-years, respectively. The COVID-19 cohort had a higher risk of ILD [adjusted hazard ratio (aHR), 11.01; 95% confidence interval (CI), 7.42–16.32] than controls. In Study 2, the vaccination cohort had a lower risk of newly diagnosed ILD than controls (aHR, 0.44; 95% CI, 0.34–0.57).

**Conclusion:**

Using nationwide data, we demonstrated that COVID-19 was associated with a higher incidence rate of newly diagnosed ILD, but that this risk could be mitigated by COVID-19 vaccination.

## Introduction

The declaration of a global coronavirus disease 2019 (COVID-19) pandemic in March 2020 followed the emergence of a novel coronavirus, severe acute respiratory syndrome coronavirus 2 (SARS-CoV-2), in December 2019 (1, 2). However, the rapid decline in COVID-19 following a surge in COVID-19 caused by the omicron variant led many governments to relax their mandates and limit population-wide intervention (3).

In this post-COVID-19 era, numerous post-COVID-19 patients continue to experience persistent respiratory symptoms such as coughing and dyspnoea, despite the absence of detectable viral infection. Some patients exhibit abnormal findings on follow-up chest computed tomography (CT) or pulmonary function tests after COVID-19 (4–7). During the COVID-19 pandemic, there was widespread concern about the subsequent development of pulmonary fibrosis after COVID-19, thus previous studies investigated pulmonary fibrosis in patients with persistent respiratory symptoms following COVID-19 (8–10). One previous meta-analysis investigated persistent lung sequelae of COVID-19 and reported that recovered patients still had chest CT abnormalities up to 1 year after infection (10).

However, to the best of our knowledge, no studies have examined the incidence of newly diagnosed interstitial lung disease (ILD) following COVID-19 using a nationwide population-based cohort. Determining the exact incidence of fibrotic sequelae caused by COVID-19 upon entering the post-COVID-19 era will aid in the long-term management of these patients. Therefore, we aimed to evaluate the association between prior COVID-19 and the incidence of newly diagnosed ILD in adults using a nationwide cohort dataset. Additionally, we assessed the impact of COVID-19 vaccination on the incidence of newly diagnosed ILD.

## Methods

### Data source

This was a retrospective cohort study using the Korean National Health Insurance claims-based dataset. The National Health Insurance Service (NHIS) is the universal insurance provider in Korea that is managed by the government and covers 97% of the Korean population (~50 million people) (11, 12). During the COVID-19 pandemic, the Korean government encouraged people to get tested for COVID-19 without delay by subsidizing the cost of diagnosis and treatment for individuals who met criteria related to COVID-19 and provided health insurance services to all Koreans with COVID-19 (NHIS-2022-1-623) (13). The NHIS database therefore includes medical data for all patients who underwent SARS-CoV-2 testing. The NHIS dataset includes demographic variables, socioeconomic characteristics, healthcare utilization (e.g., outpatient visits, emergency department visits, and hospitalization), health screening examination findings, disease diagnoses based on the 10th revision of the International Classification of Disease (ICD-10) codes, and treatments such as medication, procedures, and surgeries (11). The NHIS has been widely used in epidemiologic studies to identify risk factors for COVID-19 and post-COVID complications (14–17).

### Study population

The Korean government provided a COVID-19 study database (*N* = 8,464,242) composed of data from 561,158 subjects diagnosed with COVID-19 at least once from October 2020 to December 2021 and 7,903,084 subjects who were not diagnosed with COVID-19 during the same period (those with negative results in SARS-CoV-2 tests or those who did not undergo SARS-CoV-2 tests). These 7,903,084 subjects were selected by stratified sampling by age and sex from the entire NHIS database (*N* = ~50 million subjects) except for 561,158 subjects who were diagnosed with COVID-19.

**Figure 1:**
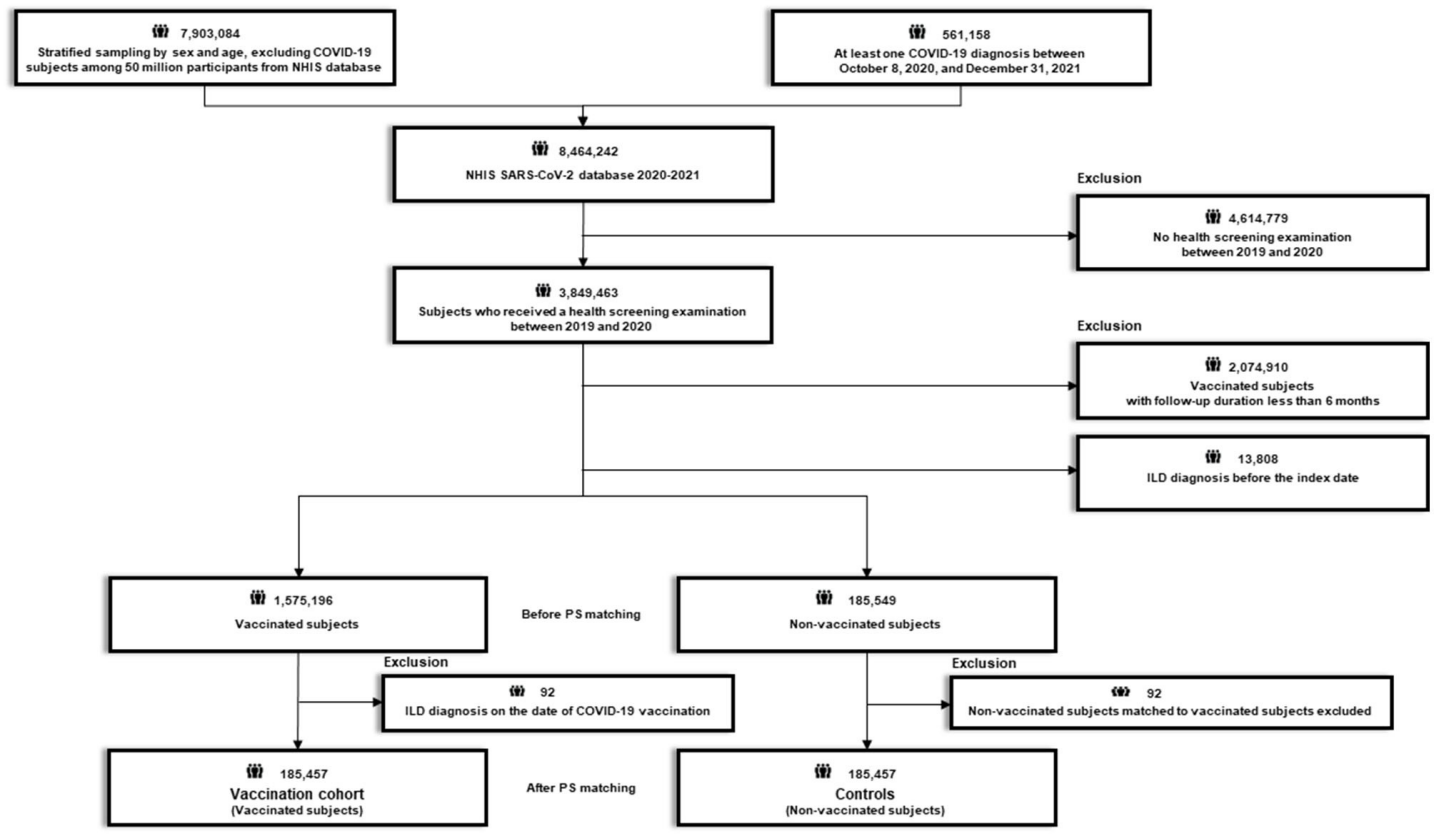
Flow chart of Study 1. COVID-19, coronavirus disease 2019; NHIS, National Health Insurance Service; SARS-CoV-2; severe acute respiratory syndrome coronavirus 2.

**Figure 2:**
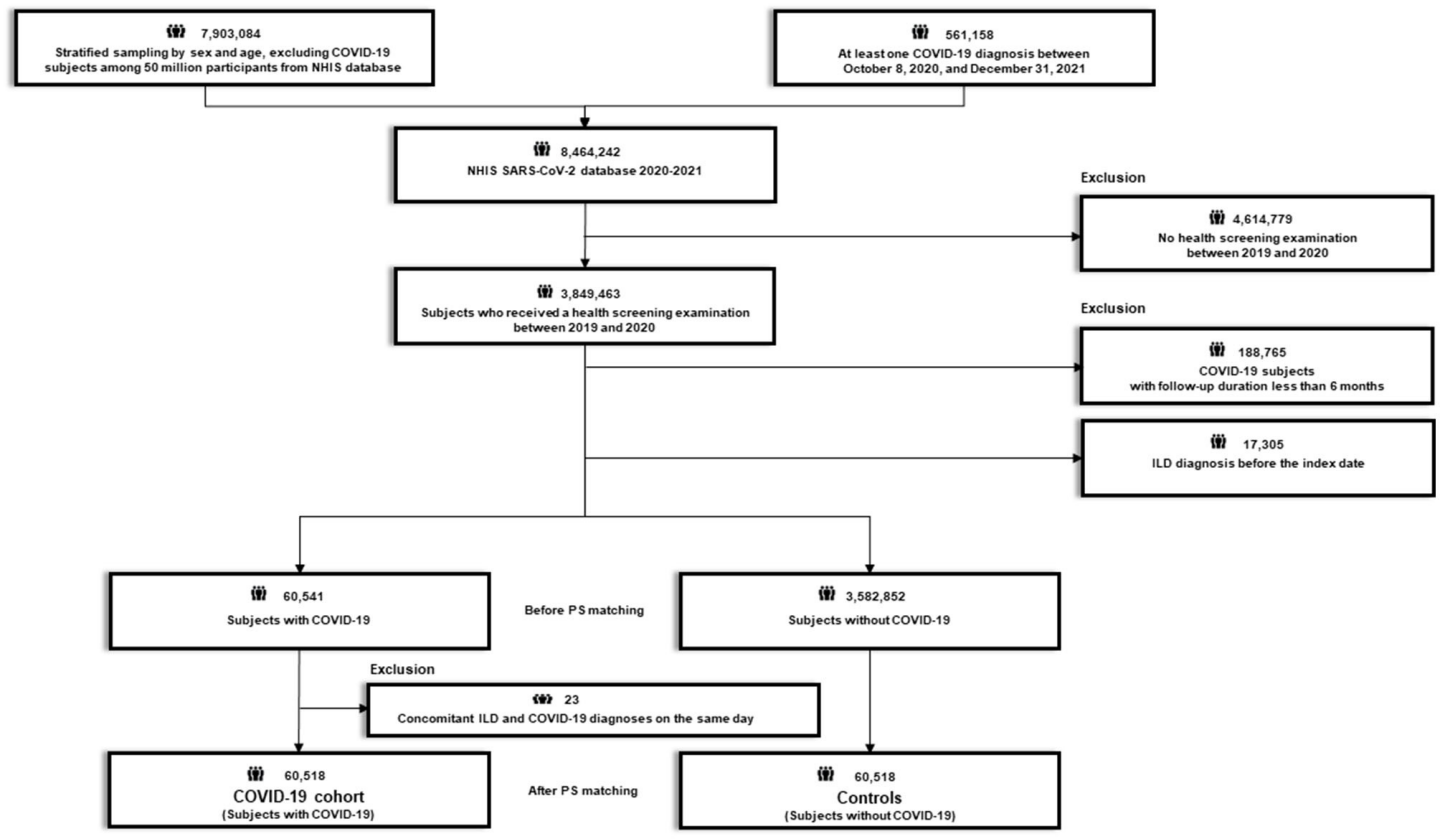
Flow chart of Study 2. COVID-19, coronavirus disease 2019; NHIS, National Health Insurance Service; SARS-CoV-2; severe acute respiratory syndrome coronavirus 2.

After excluding 4,614,779 subjects who did not undergo health screening examination between 2019 and 2020, 3,849,463 subjects who received a routine health screening examination between 2019 and 2020 were initially selected (Figures 1, 2). The recruitment period for Study 1 was from October 8, 2020, to June 30, 2021, with a 6-month follow-up period based on the date of COVID-19 confirmation. The recruitment period for Study 2 was from February 26, 2021 (COVID-19 vaccination start date) to June 30, 2021, with a 6-month follow-up period based on the date of COVID-19 vaccination.

**Study 1** aimed to evaluate whether COVID-19 is associated with an increased risk of newly diagnosed ILD by comparing the risk of newly diagnosed ILD between participants who had COVID-19 (COVID-19 cohort) and their 1:1 propensity score (PS)-matched controls. Using the 3,849,463 initial participants, we excluded 17,305 subjects with a diagnosis of ILD before the index date (COVID-19 confirmation date) and those with a follow-up duration of <6 months (*n* = 188,765). This left a total of 3,643,393 subjects; of these, 60,541 subjects had COVID-19 and 3,582,852 subjects did not have COVID-19 during the study period. Additionally, 23 subjects diagnosed with ILD concomitant with their COVID-19 diagnosis were excluded from the subjects with COVID-19. Thereafter, we performed 1:1 PS matching between subjects with and without COVID-19, and finally, 60,518 subjects with COVID-19 and 1:1 PS matched 60,518 subjects without COVID-19 (controls) were identified (Figure 1; Supplementary Figure 1A).

**Study 2** aimed to evaluate whether COVID-19 vaccination was associated with a reduced risk of newly diagnosed ILD by comparing the risk of newly diagnosed ILD between participants who were vaccinated against COVID-19 and their 1:1 PS-matched controls. Using the 3,849,463 participants, we excluded 13,808 with a diagnosis of ILD before the index date (COVID-19 vaccination date), and an additional 2,074,910 subjects with a follow-up duration of <6 months. Additionally, 92 patients diagnosed with ILD on the date of COVID-19 vaccination and their matched pairs (*n* = 92) were excluded from the vaccinated group and non-vaccinated group, respectively. Finally, 185,457 subjects who received COVID-19 vaccination (vaccination cohort) were 1:1 PS matched with 185,457 subjects who were not vaccinated (controls) (Figure 2; Supplementary Figure 1B).

Our study protocol was approved by the Institutional Review Board of Hanyang University Hospital (No. 2023-06-054). The requirement for informed consent was waived because all patient records were anonymized before use.

### Study exposure

The study exposure of Study 1 was COVID-19. Laboratory diagnosis of SARS-CoV-2 infection was defined as a positive result from real-time RT–PCR assay of nasal or pharyngeal swabs from patients with a history of SARS-CoV-2 (U071) defined using ICD-10 codes (18). The cohort entry date for each patient tested for SARS-CoV-2 was the date of the first SARS-CoV-2 test.

The study exposure of Study 2 was COVID-19 vaccination. During the study period, the NHIS provided COVID-19 vaccination data for the study purpose. The vaccination cohort included subjects who received at least one dose of the vaccine. For patients who received multiple vaccinations, the index date was set to the first vaccination date. During the study period, there were no subjects who received a third vaccination.

### Study outcome

The primary outcome was newly diagnosed ILD. ILD was defined as one or more claims under ICD-10 diagnostic code J84.x as a major or minor diagnosis (14).

### Covariates

Basic demographic characteristics of age, sex, residential area, and income status were collected from the dataset. Income status was divided into the highest 30% (high), lowest 30% (low), and the rest (middle); individuals supported by the medical aid program were classified as the low-income group. Residential areas were classified as metropolitan cities, middle- and small-sized cities, or rural areas. Smoking status (never-smoker or smoker) and alcohol consumption (none, 1–2 times a week, 3–4 times a week, and almost every day) were determined based on a self-reported questionnaire. Body mass index (BMI) was calculated as body weight divided by the square of height (kg/m^2^) and subjects were classified into four groups based on BMI as follows: normal (18.5–22.9 kg/m^2^), low (<18.5 kg/m^2^), overweight (23.0–24.9 kg/m^2^), or obese (≥ 25 kg/m^2^).

Using ICD-10 codes, the comorbidities of hypertension (I10–13, and I15), diabetes mellitus (DM, E10–14), chronic kidney disease (CKD, N18), allergic rhinitis (J30), and/or dyslipidemia (E78) with at least one diagnosis within 1 year were also recorded (12, 19–26).

### Statistical analysis

Descriptive statistics are presented as numbers (percentages) for categorical variables and mean ± standard deviations (SD) for continuous variables. We compared two groups using the χ^2^ test for categorical variables and *t*-tests for continuous variables. The incidence rates of ILD were calculated by dividing the number of incident events by the total follow-up period (1,000 person-years). A cumulative incidence plot was used to compare the incidence of ILD, and a log-rank test was used to evaluate significant differences between groups.

We performed 1:1 PS matching between the study cohort and controls based on age, sex, BMI, smoking status, alcohol consumption, economic status, residential area, and comorbidities (hypertension, DM, CKD, allergic rhinitis, and dyslipidemia). Standardized mean difference (SMD) was used to examine the balance of covariate distributions between the groups, and an SMD > 0.1 was considered to indicate an imbalance (27). Cox proportional hazards regression analyses were used to evaluate the risk of incident ILD. To further minimize potential bias that could persist even after PS matching, we additionally adjusted for age, sex, BMI, smoking status, alcohol consumption, economic status, residential area, and comorbidities (hypertension, DM, CKD, allergic rhinitis, dyslipidemia). Stratified analyses were also performed by sex, age, BMI, smoking status, alcohol consumption, economic status, residential area, and comorbidities. A two-sided *p*-value < 0.05 was considered statistically significant. All statistical analyses were performed using SAS version 9.4 (SAS Institute Inc., Cary, NC, USA).

## Results

### Baseline characteristics

The baseline characteristics of the study participants (*n* = 121,036) in Study 1 are presented in Table 1. After PS matching, there were no significant imbalances in baseline characteristics between the COVID-19 and control cohorts (all SMDs < 0.1). The mean age of the study population was 51 years and 47.9% were males.

**Table 1:** Baseline characteristics.

	**Total (*N* = 121,036)**	**COVID-19 cohort (*n* = 60,518)**	**Controls (*n* = 60,518)**	**SMD**
**Age, years**				0.062
≤ 29	10,516 (8.7)	5,077 (8.4)	5,439 (9.0)	
30–39	17,845 (14.7)	8,633 (14.3)	9,212 (15.2)	
40–49	24,351 (20.1)	12,274 (20.3)	12,077 (20.0)	
50–59	28,641 (23.7)	15,041 (24.9)	13,600 (22.5)	
60–69	26,298 (21.7)	12,926 (21.4)	13,372 (22.1)	
≥70	13,385 (11.1)	6,567 (10.9)	6,818 (11.3)	
Sex, male	57,946 (47.9)	29,460 (48.7)	28,486 (47.1)	0.032
**BMI**				0.030
Normal (18.5–22.9 kg/m^2^)	39,245 (32.4)	19,274 (31.8)	19,971 (33.0)	
Low (< 18.5 kg/m^2^)	3,485 (2.9)	1,740 (2.9)	1,745 (2.9)	
Overweight (23.0–24.9 kg/m^2^)	28,712 (23.7)	14,279 (23.6)	14,433 (23.8)	
Obese (≥25 mg/k^2^)	49,594 (41.0)	25,225 (41.7)	24,369 (40.3)	
Smoking status, smoker	37,265 (30.8)	19,120 (31.6)	18,145 (30.0)	0.035
**Alcohol consumption**				0.045
None	74,205 (61.3)	36,511 (60.3)	37,694 (62.3)	
1–2 times a week	32,649 (27.0)	16,583 (27.4)	16,066 (26.5)	
3–4 times a week	10,835 (9.0)	5,640 (9.3)	5,195 (8.6)	
Almost every day	3,347 (2.8)	1,784 (2.9)	1,563 (2.6)	
**Economic status** ^*^				0.003
Low	21,693 (17.9)	10,828 (17.9)	10,865 (18.0)	
Middle	60,502 (50.0)	30,296 (50.1)	30,206 (49.9)	
High	38,841 (32.1)	19,394 (32.0)	19,447 (32.1)	
**Residential area**				0.034
Metropolitan cities	91,701 (75.8)	45,427 (75.1)	46,274 (76.5)	
Mid-size and small cities	4,655 (3.8)	2,354 (3.9)	2,301 (3.8)	
Rural areas	24,680 (20.4)	12,737 (21.0)	11,943 (19.7)	
**Comorbidities**				
Hypertension	26,278 (21.7)	13,514 (22.3)	12,764 (21.1)	0.030
Diabetes mellitus	14,182 (11.7)	7,340 (12.1)	6,842 (11.3)	0.026
Chronic kidney disease	790 (0.7)	414 (0.7)	376 (0.6)	0.008
Allergic rhinitis	28,992 (24.0)	14,748 (24.4)	14,244 (23.5)	0.020
Dyslipidaemia	13,630 (11.3)	7,013 (11.6)	6,617 (10.9)	0.021

Data are presented as number (percentage).

COVID-19, coronavirus disease 2019; SMD, standardized mean difference; BMI, body mass index.

* Income status was divided into the highest 30% (high), the lowest 30% (low), and the rest (middle); individuals supported by the medical aid program were classified as the low-income group.

A total of 370,914 subjects were analyzed for Study 2. The baseline characteristics of study participants in Study 2 are summarized in Supplementary Table 1. There were no significant imbalances in baseline characteristics between the vaccination and control cohorts (all SMDs < 0.1).

### Incidence and risk of newly diagnosed ILD according to COVID-19

**Figure 3:**
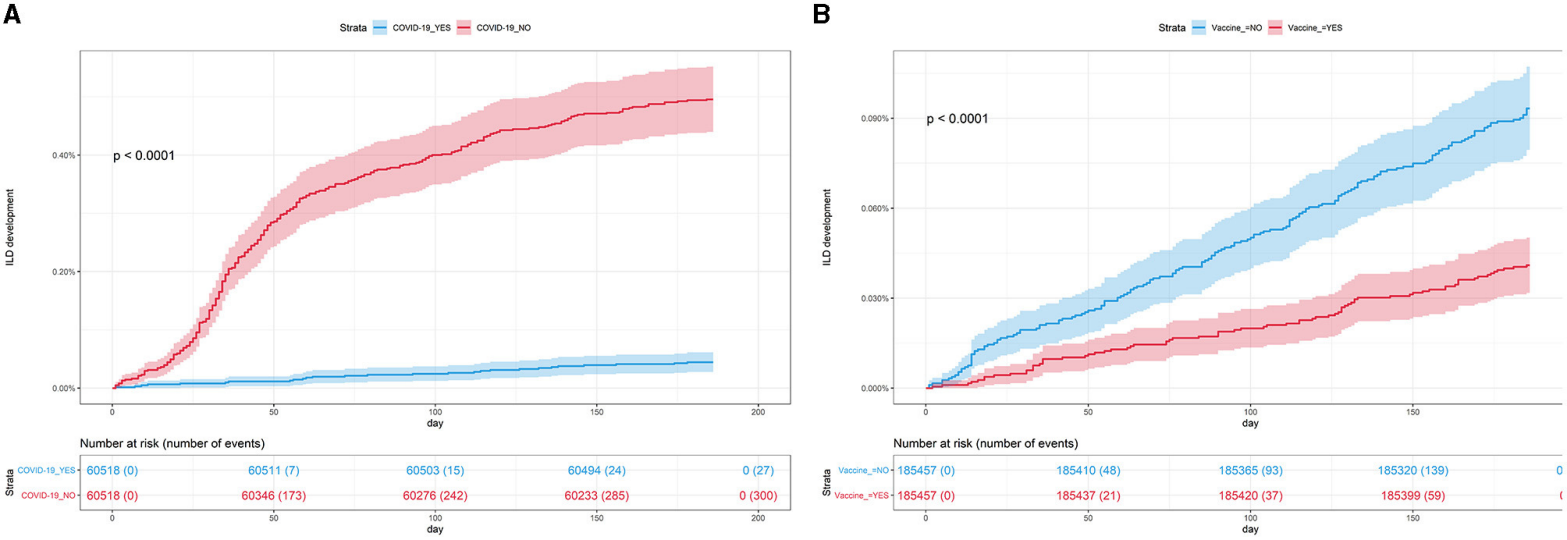
**(A)** Cumulative incidence of ILD according to COVID-19 (*n* = 121,036). **(B)** Cumulative incidence of ILD according to COVID-19 vaccination (*n* = 370,914). COVID-19, coronavirus disease 2019; ILD, interstitial lung disease.

In Study 1, the incidence and risk of newly diagnosed ILD were compared between the COVID-19 cohort and the PS-matched controls. During 6 months of follow-up, 0.50% of the COVID-19 cohort (300/60,518) and 0.04% of controls (27/60,518) developed newly diagnosed ILD, with an incidence of 9.76 and 0.88 per 1,000 person-years, respectively. Similarly, there was a significant difference in the cumulative incidence of newly diagnosed ILD between the COVID-19 cohort and controls (Figure 3A, log-rank *p* < 0.001). As shown in Table 2, the COVID-19 group had a higher risk of ILD [adjusted hazard ratio (aHR), 11.01; 95% confidence interval (CI), 7.42–16.32] than controls.

**Table 2:** Risk of ILD according to COVID-19 (Study 1).

	***N***	**Incident cases of ILD**	**Incidence per 1,000**	**Unadjusted HR (95% CI)**	Adjusted HR^*^(95% CI)
Controls	60,518	27	0.88	Reference	Reference
COVID-19 cohort	60,518	300	9.76	11.47 (7.51–16.51)	11.01 (7.42–16.32)

Data are presented as a risk ratio (95% confidence interval).

* Adjusted for age, sex, BMI, smoking status, alcohol consumption, economic status, residential area, and comorbidities (hypertension, diabetes mellitus, chronic kidney disease, allergic rhinitis, and dyslipidemia).

ILD, interstitial lung disease; COVID-19, coronavirus disease 2019; HR, hazard ratio; CI, confidence interval.

### Incidence and risk of newly diagnosed ILD according to COVID-19 vaccination

In Study 2, the incidence and risk of newly diagnosed ILD were compared between the vaccination cohort and controls. As shown in Table 3, 0.04% of the vaccination cohort (76/185,457) and 0.09% of controls (173/185,457) developed ILD, with an incidence of 0.80 and 1.83 per 1,000 person-years, respectively. Subjects in the vaccination cohort had a lower risk of ILD (aHR, 0.44; 95% CI, 0.34–0.57) than controls, consistent with the results shown in Figure 3B (log-rank *p* < 0.001).

**Table 3:** Risk of ILD according to COVID-19 vaccination (Study 2).

	***N***	**Incident cases of ILD**	**Incidence per 1,000**	**Unadjusted HR (95% CI)**	Adjusted HR^*^(95% CI)
**COVID-19 vaccination**					
No	185,457	173	1.83	Reference	Reference
Yes	185,457	76	0.80	0.44 (0.34–0.58)	0.44 (0.34–0.57)

Data are presented as risk ratios (95% confidence intervals).

**#x0002A;:** Adjusted for age, sex, BMI, smoking status, alcohol consumption, economic status, residential area, and comorbidities (hypertension, diabetes mellitus, chronic kidney disease, allergic rhinitis, and dyslipidemia).

COVID-19, coronavirus disease 2019; ILD, interstitial lung disease; HR, hazard ratio; CI, confidence interval.

### Subgroup analysis

As shown in Table 4, in both univariable and multivariable analyses, factors other than alcohol consumption did not have a significant interaction on the association between COVID-19 and ILD development (*p* for interaction > 0.05). The risk of incident ILD was different in subgroups based on the level of alcohol consumption (None, unadjusted HR 8.50, 95% CI 5.52–13.11; 1–2 times a week, unadjusted HR 18.21, 95% CI 6.66–49.77; aHR 8.37, 95% CI 5.43–12.90; 1–2 times a week, aHR 17.70, 95% CI 6.47–48.42; *p* for interaction = 0.041 for both unadjusted and adjustesd analyses).

**Table 4:** Subgroup analyses.

					**Univariable analysis**		**Multivariable analysis**	

	**COVID-19**	***N***	**Incident cases of ILD**	**Incidence per 1,000**	**Unadjusted HR**	**95% CI**	**Adjusted HR** ^*^	**95% CI**
**Age, years**								
40–49	No	12,077	3	0.49	Reference		Reference	
	Yes	12,274	23	3.68	7.55	2.27–25.14	7.61	2.28–25.36
≤ 29	No	5,439	0	0.00	Reference		Reference	
	Yes	5,077	2	0.77	–	–	–	–
30–39	No	9,212	1	0.21	Reference		Reference	
	Yes	8,633	7	1.59	7.47	0.92–60.74	7.90	0.97–64.31
50–59	No	13,600	5	0.72	Reference		Reference	
	Yes	15,041	59	7.72	10.69	4.29–26.63	10.23	4.11–25.52
60–69	No	13,372	8	1.17	Reference		Reference	
	Yes	12,926	120	18.33	15.59	7.62–31.89	15.2	7.43–31.10
≥70	No	6,818	10	2.88	Reference		Reference	
	Yes	6,567	89	26.83	9.30	4.84–17.88	9.09	4.73–17.49
*p* for interaction					0.847		0.859	
**Sex**								
Male	No	28,486	14	0.96	Reference		Reference	
	Yes	29,460	172	11.50	11.91	6.91–20.54	11.51	6.67–19.85
Female	No	32,032	13	0.80	Reference		Reference	
	Yes	31,058	128	8.11	10.18	5.75–18.00	10.26	5.80–18.16
*p* for interaction					0.695		0.758	
**BMI, kg/m** ^2^								
Normal	No	19,971	4	0.39	Reference		Reference	
	Yes	19,274	62	6.33	16.09	5.85–44.21	16.58	6.03–45.57
Low	No	1,745	2	2.25	Reference		Reference	
	Yes	1,740	4	4.52	2.01	0.37–10.95	1.64	0.30–8.99
Overweight	No	14,433	11	1.50	Reference		Reference	
	Yes	14,279	74	10.21	6.81	3.62–12.84	6.80	3.61–12.81
Obese	No	24,369	10	0.81	Reference		Reference	
	Yes	25,225	160	7.83	9.52	6.00–15.09	9.42	5.94–14.94
*p* for interaction					0.940		0.915	
**Smoking status**								
Never-smoker	No	42,373	22	1.02	Reference		Reference	
	Yes	41,398	185	8.80	8.63	5.54–13.42	8.61	5.54–13.40
Smoker	No	18,145	5	0.54	Reference		Reference	
	Yes	19,120	115	11.85	21.89	8.94–53.59	21.32	8.70–52.20
*p* for interaction					0.068		0.077	
**Alcohol consumption**								
None	No	37,694	23	1.20	Reference		Reference	
	Yes	36,511	189	10.19	8.50	5.52–13.11	8.37	5.43–12.90
1–2 times a week	No	16,066	4	0.49	Reference		Reference	
	Yes	16,583	75	8.90	18.21	6.66–49.77	17.70	6.47–48.42
3–4 times a week	No	5,195	0	0.00	Reference		Reference	
	Yes	5,640	23	8.03	–	–	–	–
Almost every day	No	1,563	0	0.00	Reference		Reference	
	Yes	1,784	13	14.38	–	–	–	–
*p* for interaction					0.041		0.041	
**Economic status**								
Low	No	10,865	3	0.54	Reference		Reference	
	Yes	10,828	73	13.30	24.49	7.72–77.72	23.91	7.54–75.89
Middle	No	30,206	13	0.84	Reference		Reference	
	Yes	30,296	122	7.92	9.37	5.29–16.61	9.21	5.20–16.31
High	No	19,447	11	1.11	Reference		Reference	
	Yes	19,394	105	10.66	9.60	5.16–17.86	9.59	5.15–17.85
*p* for interaction					0.957		0.918	
**Residential area**								
Metropolitan cities	No	46,274	23	0.98	Reference		Reference	
	Yes	45,427	225	9.80	10.03	6.53–15.41	9.83	6.4–15.10
Middle and small cities	No	2,301	0	0.00	Reference		Reference	
	Yes	2,354	14	11.70	–	–	–	–
Rural areas	No	11,943	4	0.66	Reference		Reference	
	Yes	12,737	60	9.27	14.10	5.12–38.78	14.10	5.12–38.82
*p* for interaction					0.987		0.987	
**Comorbidities**								
Hypertension	No	12,764	7	1.08	Reference		Reference	
	Yes	13,514	113	16.50	15.31	7.13–32.84	15.67	7.30–33.63
*p* for interaction					0.296		0.272	
Diabetes mellitus	No	6,842	6	1.72	Reference		Reference	
	Yes	7,340	73	19.65	11.40	4.96–26.20	11.54	5.02–26.55
*p* for interaction					0.931		0.873	
Chronic kidney disease	No	376	0	0.00	Reference		Reference	
	Yes	414	7	33.58	–	–	–	–
*p* for interaction					0.99		0.99	
Allergic rhinitis	No	14,244	12	1.65	Reference		Reference	
	Yes	14,748	88	11.76	7.10	3.89–12.98	6.80	3.72–12.44
*p* for interaction					0.085		0.08	
Dyslipidaemia	No	6,617	3	0.89	Reference		Reference	
	Yes	7,013	48	13.49	15.15	4.72–48.63	15.76	4.90–50.65
*p* for interaction					0.573		0.513	

Data are presented as hazard ratios (95% confidence intervals).

* Adjusted for age, sex, BMI, smoking status, alcohol consumption, economic status, residential area, and comorbidities (hypertension, diabetes mellitus, chronic kidney disease, allergic rhinitis, and dyslipidemia).

ILD, interstitial lung disease; COVID-19, coronavirus disease 2019; HR, hazard ratio; CI, confidence interval; BMI, body mass index.

## Discussion

Our study is the largest comprehensive study to evaluate the risk of newly diagnosed ILD in adults after COVID-19 based on analyses of nationwide cohort data. Subjects with COVID-19 had an ILD incidence rate of 9.76 per 1,000 person-years, which is 11-fold higher than that of those without COVID-19. Additionally, we analyzed the effects of COVID-19 vaccination and found that vaccination was effective at reducing the incidence of newly diagnosed ILD.

Many previous studies have reported persistent ILD after COVID-19 (5, 6, 10, 28–32); however, the number of subjects included in these studies was relatively small, and the follow-up duration was relatively short ranging from a few weeks (30) to 1 year after COVID-19 (6, 10, 28, 29, 31). For example, a Chinese study reported that 118 out of 1,279 patients underwent CT, and among these patients, 65 (55%) had persistent CT abnormalities (28). Swiss and French multicentre prospective studies reported that severe COVID-19 patients show a greater decrease in lung function and more radiologic lung sequelae than non-severe COVID-19 patients (6, 29). Although these previous studies demonstrated an association between COVID-19 and ILD, the actual estimated incidence and risk of clinically relevant ILD following COVID-19 in real-world clinics has not been reported. Our results indicate that the risk of ILD is about 11-fold higher in those with COVID-19 than those without COVID-19. Notably, the cumulative curve showed a steep increase in the slope until about 2 months after infection, but ILD incidence showed a steady increase even after 2 months. Based on the results of our study, we recommend that clinicians perform follow-up chest CTs or pulmonary function tests in post-COVID-19 patients over a long period to detect abnormalities in the lung interstitium, especially in those patients with persistent respiratory symptoms (e.g., cough and dyspnoea).

Another important finding of our study is that COVID-19 vaccination can decrease the risk of post-COVID-19 ILD at the population level, demonstrating the preventive role of COVID-19 vaccination on this disastrous post-COVID-19 complication. However, at the individual level, there have been some concerns that the COVID-19 vaccine could cause or aggravate ILD (33–36). Although some cases were successfully recovered with corticosteroid treatments (33–35), one study in which 37% had underlying pre-diagnosed ILD, reported the mortality rate was 15% (35). Accordingly, based on the findings of a previous study that has demonstrated the safety of the vaccine (37) and our research, maintaining a positive position on COVID-19 vaccination against post-COVID-19 ILD, a balanced understanding of the relationship between COVID-19 vaccination and ILD risk, is needed.

ILD may develop after COVID-19 for several reasons. First, post-infectious organizing status or ARDS-related fibrosis should be considered in patients with severe COVID-19 pneumonia and ARDS (38). Ventilator-induced lung injury could also be associated with post-infection fibrotic changes. However, ILD may occur without prior COVID-19 acute respiratory distress syndrome or lung injury from mechanical ventilation during treatment of severe COVID-19. One study delineated immune-proteomics in the airway and peripheral blood of healthy controls and post-COVID-19 patients 3–6 months after discharge (39). The authors of that study reported that increased B cell numbers and altered monocyte subsets were associated with widespread lung abnormalities. Another earlier study reported that an increase in the frequency of airway and lung B cells similar to that seen in the airway after COVID-19 was also present in interstitial pulmonary fibrosis (IPF), a representative ILD (40). Thus, in the post-COVID-19 airway, B cells may be directly promoting aberrant lung tissue repair (41). Peripheral blood mononuclear cell signatures in COVID-19 lung disease and IPF have also been shown to exhibit comparable transcriptional signatures and have prognostic value (42). There is also evidence that monocyte and T cell subsets are involved in the pathogenesis of post-COVID-19 ILD; a recent study evaluating blood samples from subjects with post-COVID-19 ILD, IPF, and controls showed that survivors of post-COVID-19 ILD had higher expression of genes related to naïve and memory CD4 T cells, Tregs, memory CD8 T GZMB+, memory CD8 T GZMK+, and naïve CD8 T cells, but lower levels in IPF, suggesting most subjects with post-COVID-19 ILD have partially or completely resolved pulmonary fibrosis, while most patients with IPF have progressive disease (43). Further studies are still needed to determine the exact mechanism(s) underlying ILD after COVID-19, but the results of previous studies suggest that the complex immune response to the virus itself may promote the development of ILD after COVID-19.

Several limitations to our study should be acknowledged. First, it is important to note that our study utilized a dataset specifically derived from the Korean population, which potentially restricts the generalizability of our findings to other countries or ethnic groups. Second, the identification of ILD and other comorbidities relied on the use of ICD-10 codes, so there might have been over- or underestimation of the diagnosis. Additionally, we could not classify the subtypes of ILD. Third, due to a lack of laboratory data and pulmonary function test data, these data could not be incorporated into our analyses. Fourth, we are unable to provide post-COVID-19 ILD outcomes related to the delta or omicron variants, since our study period (from October 2020 to June 2021) was performed before the delta or omicron variants dominated (major variant changes in Korea are as follows: alpha and beta variants from December 2020, gamma variant from January 2021, delta variant from May 2021, and omicron variant from November 2021). Further study of post-COVID-19 ILD associated with delta or omicron variants will be required. An additional limitation is that due to the relatively short follow-up duration of our dataset, we were unable to provide results with more than 1 year of follow-up. Thus, further study of longer follow-up is needed. Finally, the exact mechanism of the effect of the COVID-19 vaccine on the prevention of newly diagnosed ILD after COVID-19 cannot be determined through our study. Future research is required to explore the mechanisms behind the increased risk of post-COVID-19 ILD and the protective role of different types of COVID-19 vaccines against post-COVID-19 ILD.

In conclusion, based on analyses of a nationwide dataset, we demonstrated that COVID-19 is associated with a higher incidence rate of newly diagnosed ILD. Additionally, we suggest that COVID-19 vaccination reduces the risk of developing ILD by preventing COVID-19 itself.

## Data availability statement

The raw data supporting the conclusions of this article will be made available by the authors, without undue reservation.

## Ethics statement

The studies involving humans were approved by the Institutional Review Board of Hanyang University Hospital (No. 2023-06-054). The studies were conducted in accordance with the local legislation and institutional requirements. The Ethics Committee/Institutional Review Board waived the requirement of written informed consent for participation from the participants or the participants' legal guardians/next of kin because all patient records were anonymized before use.

## Author contributions

B-GK: Conceptualization, Data curation, Methodology, Writing—original draft, Writing—review & editing. HL: Conceptualization, Data curation, Methodology, Validation, Writing—original draft, Writing—review & editing. CJ: Conceptualization, Data curation, Formal analysis, Methodology, Visualization, Writing—review & editing. SY: Conceptualization, Data curation, Formal analysis, Methodology, Visualization, Writing—review & editing. DP: Conceptualization, Data curation, Writing—review & editing. TP: Conceptualization, Data curation, Writing—review & editing. J-YM: Conceptualization, Data curation, Writing—review & editing. T-HK: Conceptualization, Data curation, Writing—review & editing. JS: Conceptualization, Data curation, Writing—review & editing. HY: Conceptualization, Data curation, Writing—review & editing. JK: Conceptualization, Data curation, Formal analysis, Methodology, Supervision, Writing—review & editing. S-HK: Conceptualization, Data curation, Methodology, Supervision, Writing—original draft, Writing— review & editing.
