# Supplementary material for: Risk of newly diagnosed interstitial lung disease after COVID-19 and impact of vaccination: a nationwide population-based cohort study
Source: Front Public Health. 2024 Jan 8;11:1295457. doi: 10.3389/fpubh.2023.1295457 (PMC10801741; doi:10.3389/fpubh.2023.1295457)
Supplement: Supplementary file 1 [file Data_Sheet_1.docx]

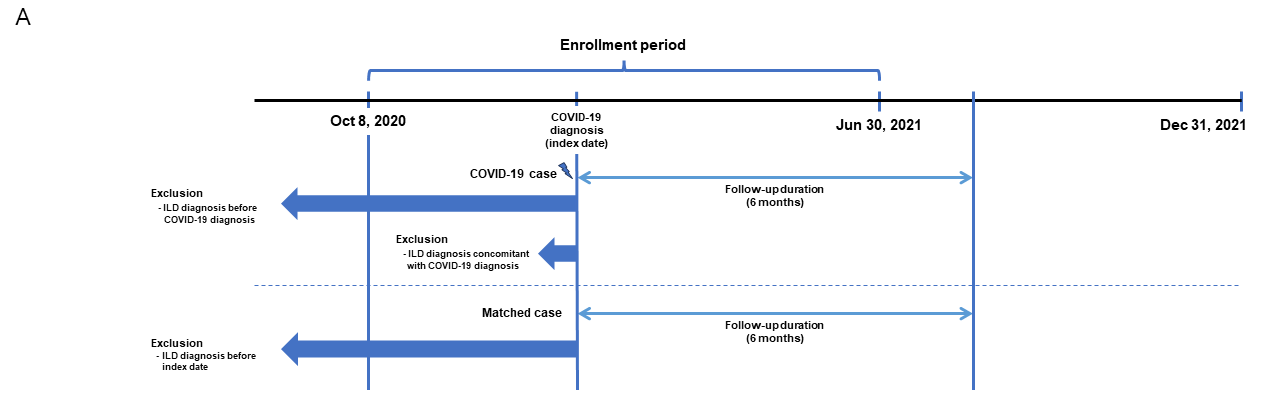


**
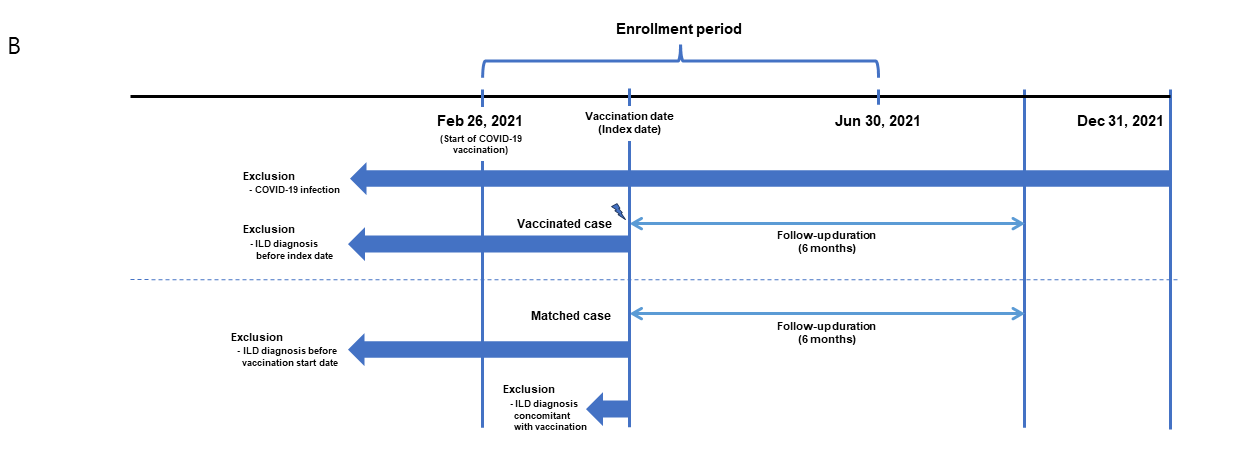
**

**Supplementary Figure 1.** schematic diagram of research design of (A) Study 1 and (B) Study 2

**Supplementary Table 1.** Baseline characteristics of patients in Study 2

|  | **Total**  **(N = 370,914)** | **COVID-19 vaccination cohort**  **(n = 185,457)** | **Control cohort**  **(n = 185,457)** | **SMD** |
| --- | --- | --- | --- | --- |
| Age, years |  |  |  | 0.019 |
| ≤ 29 | 53,202 (14.3) | 26,710 (14.4) | 26,492 (14.3) |  |
| 30 - 39 | 105,032 (28.3) | 51,813 (27.9) | 53,219 (28.7) |  |
| 40 - 49 | 77,468 (20.9) | 38,859 (21.0) | 38,609 (20.8) |  |
| 50 - 59 | 51,787 (14.0) | 26,279 (14.2) | 25,508 (13.8) |  |
| 60 - 69 | 51,219 (13.8) | 25,780 (13.9) | 25,439 (13.7) |  |
| ≥ 70 | 32,206 (8.7) | 16,016 (8.6) | 16,190 (8.7) |  |
| Sex, male | 176,429 (47.6) | 86,723 (46.8) | 89,706 (48.4) | 0.032 |
| BMI |  |  |  | 0.059 |
| Normal (18.5–22.9 kg/m2) | 149,038 (40.2) | 75,214 (40.6) | 73,824 (39.8) |  |
| Low (< 18.5 kg/m2) | 21,934 (5.9) | 10,015 (5.4) | 11,919 (6.4) |  |
| Overweight (23.0–24.9 kg/m2) | 78,953 (21.3) | 40,864 (22.0) | 38,089 (20.5) |  |
| Obese (≥ 25 mg/k2) | 120,989 (32.6) | 59,364 (32.0) | 61,625 (33.2) |  |
| Smoking status, smoker | 119,180 (32.1) | 57,571 (31.0) | 61,609 (33.2) | 0.047 |
| Alcohol consumption |  |  |  | 0.040 |
| None | 253,272 (68.3) | 128,155 (69.1) | 125,117 (67.5) |  |
| 1 - 2 times a week | 80,133 (21.6) | 39,376 (21.2) | 40,757 (22.0) |  |
| 3 - 4 times a week | 27,174 (7.3) | 13,145 (7.1) | 14,029 (7.6) |  |
| Almost every day | 10,335 (2.8) | 4,781 (2.6) | 5,554 (3.0) |  |
| Economic status^*^ |  |  |  | 0.028 |
| Low | 69,630 (18.8) | 34,109 (18.4) | 35,521 (19.2) |  |
| Middle | 198,254 (53.5) | 98,792 (53.3) | 99,462 (53.6) |  |
| High | 103,030 (27.8) | 52,556 (28.3) | 50,474 (27.2) |  |
| Residential area |  |  |  | 0.010 |
| Metropolitan cities | 268,209 (72.3) | 134,357 (72.4) | 133,852 (72.2) |  |
| Mid-size and small cities | 21,044 (5.7) | 10,324 (5.6) | 10,720 (5.8) |  |
| Rural areas | 81,661 (22.0) | 40,776 (22.0) | 40,885 (22.0) |  |
| Comorbidities |  |  |  |  |
| Hypertension | 54,229 (14.6) | 26,804 (14.5) | 27,425 (14.8) | 0.009 |
| Diabetes mellitus | 31,157 (8.4) | 15,043 (8.1) | 16,114 (8.7) | 0.021 |
| Chronic kidney disease | 3,736 (1.0) | 1,857 (1.0) | 1,879 (1.0) | 0.001 |
| Allergic rhinitis | 74,477 (20.1) | 37,252 (20.1) | 37,225 (20.1) | < 0.001 |
| Dyslipidemia | 28,609 (7.7) | 13,881 (7.5) | 14,728 (7.9) | 0.017 |

Data are presented as number (percentage).

***Abbreviations:*** COVID-19, coronavirus disease 2019; SMD, standardized mean difference; BMI, body mass index.

^*^Income status was divided into the highest 30% (high), the lowest 30% (low), and the rest (middle); individuals supported by the medical aid program were classified as the low-income group.
